# Resveratrol as an Adjunctive Therapy for Excessive Oxidative Stress in Aging COVID-19 Patients

**DOI:** 10.3390/antiox10091440

**Published:** 2021-09-09

**Authors:** Min-Tser Liao, Chia-Chao Wu, Shu-Fang Vivienne Wu, Mei-Chen Lee, Wan-Chung Hu, Kuo-Wang Tsai, Chung-Hsiang Yang, Chien-Lin Lu, Sheng-Kang Chiu, Kuo-Cheng Lu

**Affiliations:** 1 Department of Pediatrics, Taoyuan Armed Forces General Hospital, Taoyuan City 325, Taiwan; liaoped804h@yahoo.com.tw (M.-T.L.); lazyalways@gmail.com (C.-H.Y.); 2 Department of Pediatrics, Taoyuan Armed Forces General Hospital Hsinchu Branch, Hsinchu City 300, Taiwan; 3 Department of Pediatrics, Tri-Service General Hospital, National Defense Medical Center, Taipei 114, Taiwan; 4 Department of Internal Medicine, Division of Nephrology, Tri-Service General Hospital, National Defense Medical Center, Taipei 114, Taiwan; wucc@mail.ndmctsgh.edu.tw; 5 Department and Graduate Institute of Microbiology and Immunology, National Defense Medical Center, Taipei 114, Taiwan; 6 School of Nursing, National Taipei University of Nursing and Health Sciences, Taipei 112, Taiwan; shufang@ntunhs.edu.tw (S.-F.V.W.); mclee@ntunhs.edu.tw (M.-C.L.); 7 Department of Medical Research, Taipei Tzu Chi Hospital, Buddhist Tzu Chi Medical Foundation, New Taipei City 231, Taiwan; wanchung.hu09@gmail.com (W.-C.H.); tch33225@tzuchi.com.tw (K.-W.T.); 8 Division of Nephrology, Department of Medicine, Fu Jen Catholic University Hospital, School of Medicine, Fu Jen Catholic University, New Taipei 24352, Taiwan; janlin0123@gmail.com; 9 Division of Infectious Diseases, Department of Medicine, Taipei Tzu Chi Hospital, Buddhist Tzu Chi Medical Foundation, New Taipei City 231, Taiwan; csk33kimo@hotmail.com; 10 Division of Infectious Diseases, Department of Medicine, Tri-Service General Hospital, National Defense Medical Center, Taipei 114, Taiwan; 11 Division of Nephrology, Department of Medicine, Taipei Tzu Chi Hospital, Buddhist Tzu Chi Medical Foundation, New Taipei City 231, Taiwan

**Keywords:** adaptive immunity, aging, antioxidants, inflammation, innate immunity, oxidative stress, resveratrol, SARS-CoV-2

## Abstract

The coronavirus disease 2019 (COVID-19) pandemic continues to burden healthcare systems worldwide. COVID-19 symptoms are highly heterogeneous, and the patient may be asymptomatic or may present with mild to severe or fatal symptoms. Factors, such as age, sex, and comorbidities, are key determinants of illness severity and progression. Aging is accompanied by multiple deficiencies in interferon production by dendritic cells or macrophages in response to viral infections, resulting in dysregulation of inflammatory immune responses and excess oxidative stress. Age-related dysregulation of immune function may cause a more obvious pathophysiological response to SARS-CoV-2 infection in elderly patients and may accelerate the risk of biological aging, even after recovery. For more favorable treatment outcomes, inhibiting viral replication and dampening inflammatory and oxidative responses before induction of an overt cytokine storm is crucial. Resveratrol is a potent antioxidant with antiviral activity. Herein, we describe the reasons for impaired interferon production, owing to aging, and the impact of aging on innate and adaptive immune responses to infection, which leads to inflammation distress and immunosuppression, thereby causing fulminant disease. Additionally, the molecular mechanism by which resveratrol could reverse a state of excessive basal inflammatory and oxidative stress and low antiviral immunity is discussed.

## 1. Introduction

The emerging novel coronavirus disease 2019 (COVID-19), which is caused by severe acute respiratory syndrome coronavirus 2 (SARS-CoV-2) infection, has burdened health systems around the world. Identifying potential risk factors and predicting disease progression may be very useful for healthcare professionals to effectively classify patients, provide personalized treatment, monitor clinical progress, and allocate appropriate resources at all levels of care to reduce morbidity and mortality [1].

Elderly people are very sensitive to viral infections; thus, disease severity and subsequent mortality are both higher in them than in younger people. COVID-19 in elderly people generally results in a high viral load, impaired virus clearance, high oxidative stress, increased inflammation, and inflammatory cytokine production [2]. In severe cases, SARS-CoV-2 induces the release of cytokines and chemokines, such as interleukin (IL), interferon (IFN), and tumor necrosis factor (TNF) at a higher rate than normal (known as a “cytokine storm”), which can cause cytopathic effects and lead to organ failure [3]. Although treatment to reduce the basic reproductive potential (R0) of the virus is essential, a search for specific treatments should not be ignored. The treatment of severely ill patients is supportive of this new type of coronavirus. However, effective therapies that are inexpensive, non-toxic, and readily available are urgently needed. Resveratrol (3, 5, 4/-trihydroxytrans-stilbene; RSV) is a naturally-occurring polyphenol compound found in grapes, red wine, blackberries, and groundnuts. RSV has received attention because of its therapeutic effects. In this regard, it has antioxidant, antitumor, antiviral, and free radical scavenging properties; thus, it can be regard as a potential adjuvant therapy [4,5]. RSV modulates the inflammatory response in a pleiotropic manner, can scavenge free radicals such as superoxide, and may interfere with infections by altering numerous cell defense pathways. [3,6]. 

The host inflammatory response is a crucial determinant of disease outcome and is correlated with disease severity in SARS-CoV-2 infections. Mechanistically, RSV can activate nuclear factor erythroid 2 p45-related factor 2 (Nrf2) [7]. In this review, we first provide a brief overview of the pathogenic mechanism of SARS-CoV-2 and then discuss in detail how aging affects immunity and changes in oxidative stress. Finally, we review the potential benefits of RSV as a dietary supplement in elderly patients with COVID-19 [8].

## 2. Hyperinflammation in COVID-19

**Figure 1:**
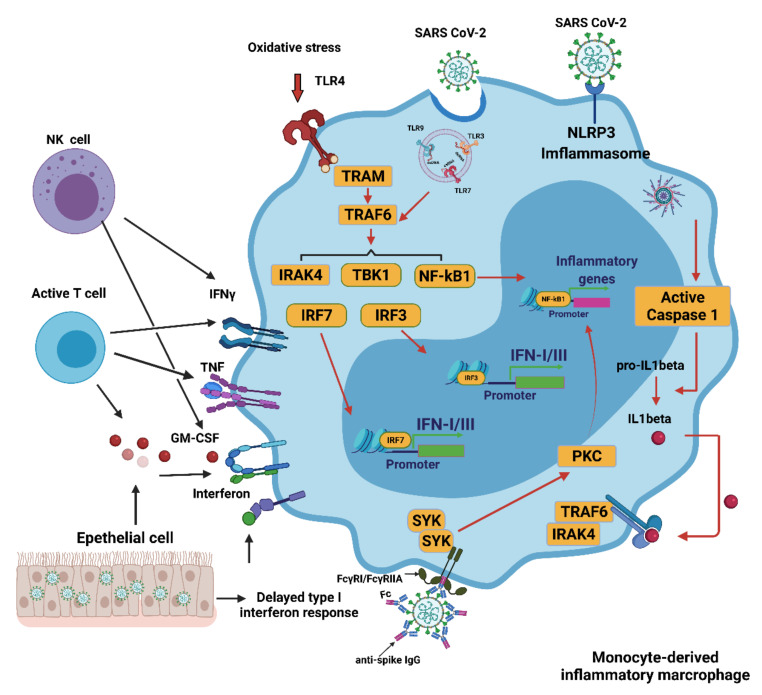
Activation of monocyte-derived macrophages in COVID-19. Several mechanisms induce the excessive activation of monocytes/macrophages during a SARS-CoV-2 infection. The delayed production of IFN I results in the continued conscription of circulating monocytes into the pulmonary parenchyma. Activated NK and T cells also favor infiltrating cells derived from monocytes. Virus detection may trigger the activation of TLR7 as a result of the recognition of the viral single-stranded RNA. IFN I increases the expression of the SARS-CoV-2 entry receptor ACE2, allowing the virus to enter macrophages and activate cytoplasmic inflammation through NLRP3. The combination of the immune complex containing the anti-spike protein IgG with the Fcγ receptor (FcγR) on activated macrophages further promotes aberrant viral entry and causes an inflammatory cascade. CCL: CC-chemokine ligand; CXCL10: CXC-chemokine ligand 10; ISG: interferon-stimulated gene; ITAM: immune 06receptor tyrosine-based activation motif; TRAM: TRIF-related adaptor molecule; NK, natural killer. Figure generated with Biorender (https://biorender.com/ accessed on 6 September 2021).

In COVID-19, multiple mechanisms can lead to the excessive activation of monocyte-derived macrophages. The delayed production of type I interferon (IFN I) leads to aggravated cellular pathological injury and the increased synthesis and secretion of mononuclear chemoattractants from alveolar epithelial cells, which leads to the continuous recruitment of blood monocytes to the lungs [9]. Infection with SARS-CoV-2 may trigger the JAK/STAT inflammatory pathway, which causes the differentiation of monocytes into pro-inflammatory macrophages and contributes to the development of a cytokine storm [10]. Recruitment and switch of monocyte-derived macrophages can be achieved by secreting granulocyte-macrophages colony stimulant factor (GM-CSF), tumor necrosis factor (TNF), and interferon-γ (IFNγ) from activated natural killer (NK) cells and T cells [11]. The recognition of oxidized products such as oxidized phospholipids (OxPLs) in the infected lung can further facilitate the activation of macrophages to secrete pro-inflammatory cytokines and chemokines through the toll-like receptor 4 (TLR4)-tumor necrosis factor receptor associated factor 6 (TRAF6)-nuclear factor κ-light-chain-enhancer of activated B cells (NF-κB) pathway [12]. The single-stranded RNA virus can be detected by LRT7 resulting in its activation. SARS-CoV-2 can cause neutrophils to release neutrophil extracellular traps (NETs). However, the NETs triggered by SARS-CoV-2 depend on angiotensin-converting enzyme 2 (ACE2), various proteases, peptidylarginine deiminase 4 (PAD4), and viral replication. NETs released by neutrophils can also promote lung epithelial cell death by increasing oxidative stress [13]. IFN I can induce the expression of ACE2 for SARS-CoV-2 entry into the cytoplasm of activated macrophages and further activate the Nod-like receptor (NLR) family pyrin domain containing 3 (NLRP3) inflammasome, leading to the secretion of either mature IL-1β, IL-18, or both. IL-1 β may amplify the activation of monocyte-derived macrophages in an autocrine or paracrine manner but may also reduce the production of IFN I in the infected lung [14]. In addition, anti-SARS-CoV-2 IgG antibodies are produced in B cells, but they can induce an antibody-dependent enhancement mechanism (ADE). This occurs with both sub- and non-neutralizing antiviral antibodies, resulting in the severity of the inflammation and oxidative stress being exacerbated. In vitro modeling of ADE has attributed the increased pathogenesis to viral entry of the Fcγ receptor (FcγR). The combination of the FcγR and the IgG immune complex of the anti-spike protein can significantly increase the number of inflammatory macrophages [15]. These activated macrophages contribute to the oxidative stress and cytokine storm seen in a COVID-19 infection. These activated macrophages then release large quantities of pro-inflammatory cytokines and chemokines [16] (Figure 1).

### 2.1. Activated Monocytes and Hypercoagulability

Circulating pro-inflammatory stimuli, such as pathogen-associated molecular patterns (PAMPs) derived from microorganisms, damage-associated molecular patterns (DAMPs) derived from injured host cells, and various cytokines trigger the activity of blood monocytes. It has been suggested that inhibiting DAMPS may be a therapeutic approach to suppress the hyperinflammatory processes seen in COVID-19 [17]. Vascular endothelial cells are activated by both cytokines and viral particles. This results in an increase in the production of monocyte chemoattractants and adherence molecules. Virus-induced endothelial damage also results in the expression of tissue factor [18]. Activated monocytic cells are recruited to the damaged endothelium and secreted exosomes combine with the high levels of expression of tissue factor on endothelial cells, which promotes fibrin deposition and coagulation. Neutrophils can also be recruited by damaged endothelial cells and constructed NETs, which activate platelets and the clotting cascade to promote blood clotting [13]. Numerous factors, such as microvascular lesions, thrombosis, anti-phospholipid antibodies, and coagulopathy, are associated with the pathological changes seen in venous and arterial thromboembolism in severe COVID-19 cases [19,20]. In patients with severe COVID-19, the blood levels of D-dimer are also significantly increased [20,21]. NETs also induce immune-related blood clotting, microvascular coagulation, and fibrin genesis via the recruitment and activation of platelets [22]. Activated platelets interact with neutrophils through P-selectin and its ligand to promote NETs formation [23]. Depletion of neutrophils can prevent thrombus formation.

### 2.2. Endothelial Cell (EC) Infection in COVID-19

SARS-CoV-2 infects the host through its receptor angiotensin 2 converting enzyme (ACE2), which is expressed in multiple organs, including the lung, heart, kidneys, and intestine. ACE2 is also expressed on endothelial cells [24]. Varga et al. found evidence of direct SARS-CoV-2 infection of endothelial cells in multiple organs and widespread endothelial inflammation associated with apoptosis [25]. The viral infection of endothelial cells results in a state of endotheliitis, in which there is a significant accumulation of inflammatory cells, and substantial death of endothelial and inflammatory cells. Damage to endothelial cells results in the strong activation of the coagulation system, as a result of the exposure of tissue factor and the activation of other pathways. Endothelial dysfunction refers to a systemic state in which the endothelium loses its physiological properties, including the tendency to promote vasodilation, fibrinolysis, and anti-aggregation [26]. Narrowing of arteries feeding organs and microcirculatory disturbances of the liver, spleen, and kidneys in patients with severe COVID-19 were described recently [27]. A histological analysis of lung vessels in COVID-19 patients revealed that capillary alveolar microthrombi were nine times more common in COVID-19 patients than in influenza patients. In lungs from patients with COVID-19, the extent of new vessel growth, predominantly through a mechanism of intussusceptive angiogenesis, was 2.7 times as high as that in patients with influenza [28]. However, as the groups were sampled at different stages of the illness, the relevance of these findings is not clear. Therefore a concluding statement of the study, which stated that “vascular angiogenesis distinguished pulmonary pathogen biology from COVID-19 from infection with the equally severe influenza virus,” should be considered speculative [29].

With respect to COVID-19, the origin of endothelial dysfunction remains unclear. The presence of direct infection of EC with SARS-CoV-2, results in endothelial damage [30]. Endothelia in the arteries and veins seem to be very susceptible to SARS-CoV-2 infection. Both arterial and venous ECs and arterial smooth muscle cells express ACE2, the receptor involved in SARS-CoV-2 infection [31], thereby placing the whole vascular system at risk of injury. However, endothelial dysfunction could also occur secondarily to the activation of either inflammatory, coagulation and complement cascades, or in combination [32]. As a result, the endothelial lesions caused by COVID-19 are at the crossroads of hypercoagulation, fibrinolysis impairment, complement system activation, and glycocalyx layer degradation [33].

Previous study reported that 2-4% of patients with acute pulmonary embolism will develop chronic thromboembolic pulmonary hypertension within 2 years of the embolic event, but this is often under-diagnosed [34]. It is currently unknown which potential sequelae, in the lungs or other organ, may arise over the long term in survivors of SARS-CoV-2 [35], but it should be the focus of future studies. In previous coronavirus epidemics, respiratory deterioration, as measured by imaging and assessments of lung-function, were observed to persist for up to 1 year after the apparent clinical recovery of the patients [36]. Therefore, it is likely to be similar in survivors of COVID-19, and it is likely that significant pulmonary vascular alterations may appear in the short to medium term after recovery from the acute infection. In addition, COVID-19 accelerates endothelial dysfunction and nitric oxide deficiency [37]. Resveratrol (RSV) increases the production of nitric oxide (NO) in endothelial cells by regulating the expression of NO endothelial synthase (eNOS), stimulating eNOS enzymatic activity, and preventing eNOS uncoupling. At the same time, RSV inhibits the synthesis of endothelin-1 and reduces oxidative stress in endothelial cells and smooth muscle cells. The proliferation of smooth muscle cells, induced by pathological stimuli, vascular re-shaping, and arterial rigidity, can also increase via treatment with RSV [38].

## 3. SARS-CoV-2 Infection and Oxidative Stress

Recent studies have suggested that oxidative stress is a key factor in the pathogenesis of COVID-19 [39,40]. Binding of SARS-CoV-2 to host cell membrane ACE2 facilitates virus entry into the host cell, thereby leading to a reduction in bioavailable ACE2. Because of the protective role of ACE2, a decrease in its level is associated with subsequent undesirable clinical phenotypes through activation of the NLRP3 inflammasome cascade [41]. The high ratio of neutrophils to lymphocytes in critically ill patients with COVID-19 is associated with excessive levels of reactive oxygen species (ROS) [42]. This oxidative stress can also be linked to ACE2. Whenever there is a dysfunction in ACE2 or when its levels are reduced due to SARS-CoV-2 infection, the overexpression of angiotensin II (Ang II) becomes a powerful pro-oxidant system in vessels and mononuclear cells [43]. Ang II has been demonstrated to bind to the type 1 angiotensin receptor (AT1R) and activate nicotinamide adenine dinucleotide phosphate (NADPH) oxidase (NOX) [44,45,46,47]. NOX activation contributes to the excessive production of ROS, such as superoxide radical anions (O_2_^•−^) and hydrogen peroxide (H_2_O_2_). This AngII mediated activation of AT1R signaling turns on NOX and induces oxidative stress and inflammation, resulting in severe COVID-19 symptoms [48].

The ROS generated by NOX reduce the bio-available nitric oxide and cause inflammation, vasoconstriction, a redox imbalance, and endothelial dysfunction [49,50]. ROS also contributes to the overexpression of NF-kB and thioredoxin interaction/inhibitory protein (TXNIP). NF-κB enhances the expression of NLRP3, pro-IL-1β, and pro-IL-18. TXNIP regulates the assembly of the NLRP3 inflammasome, which contributes to severe inflammation [16]. Therefore, the cytopathic effects of SARS-CoV-2 may result in pyroptosis [51,52], an inflammatory form of cell death elicited by inflammasomes, which leads to the breakdown of gasdermin D (GSDMD) and activation of inactive cytokines such as IL-18 and IL-1β. Violi et al. demonstrated that NADPH oxidase-2 (NOX-2) is amplified in hospitalized COVID-19 patients [53].

The release of iron from red blood cells into the blood stream is another source of ROS in COVID-19 patients. This pathogenesis occurs because SARS-CoV-2 can damage hemoglobin (Hb) in the RBCs, thereby eliciting the release of free Fe(III) ions from affected heme into the circulation [54]. This in turn increases serum ferritin levels [20]. Therefore, SARS-CoV-2-mediated hemoglobinopathy and a dysregulation of iron metabolism contribute to ferroptosis, oxidative stress, lipid and protein peroxidation, and mitochondrial injury [55].

## 4. Innate Immune Response to COVID-19

### 4.1. Innate Immune Response

The innate immune response to respiratory pathogens is a highly ordered process, involving several different layers of defense [56]. In respiratory infections, respiratory epithelial cells, mast cells, and macrophages can detect invasive pathogens [57]. PAMPs combine with host sensor cell membrane pattern recognition receptors (PRRs), such as toll-like receptors (TLRs) and retinoic acid-inducible gene I (RIG-I) receptors, and adaptive molecules, resulting in an immune response tailored to the pathogen [58]. PAMPs trigger an association between PRRs and adaptor molecules, leading to an immune reaction. Endosomal PRRs include toll-like receptors (TLRs) 3, 7, and 8, which recognize extracellular PAMPs such as viral RNA [59]. Cytoplasmic RNA sensors such as RIG-I [58] and MDA5 (melanoma differentiation-associated protein 5) can also bind to viral double-stranded RNA (dsRNA) [60].

Intracellular DAMPs released from dead cells [61] initiate PRRs in epithelial cells to trigger pro-inflammatory cytokine release [62]. Thereafter, more effective mononuclear cells and T lymphocytes are attracted to further drive the inflammatory process.

Detecting coronavirus through RIG-I and other PRRs triggers an obvious innate immune response, which would effectively limit viral replication [62]. First, IFN-I (IFN-α and IFN-β) or IFN-III cytokines are released to promote adequate eradication of the virus [63]. Secreted IFN activates IFN-stimulated genes (ISGs) that exert direct antiviral properties and recruit effective antiviral immune cells such as myeloid cells [64].

### 4.2. Interferons (IFNs), Proinflammatory Cytokines, and Oxidative Stress

IFNs are important antiviral cytokines [63] and almost all nucleated cells respond to IFN I. The response to IFN III is restricted to the barrier of the respiratory tract or digestive tract [65], but long-term treatment with IFN-III in viral infections may be harmful [66]. 

**Figure 2:**
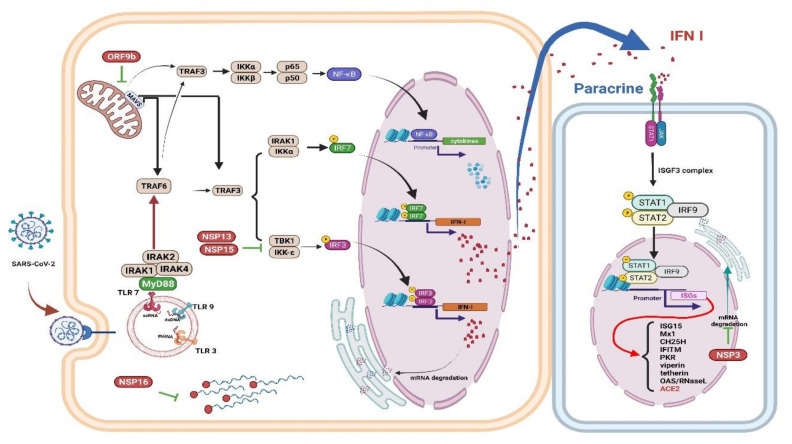
Immune evasion by SARS-CoV-2. The schematic shows how SARS-CoV-2 viral proteins are able to inhibit various immune processes such as pathogen recognition, IFN production and signaling, and ISGs [76]. Studies have shown that each viral protein can block different key signaling cascades. Viral RNA can be assembled with guanosine and methylated at the 5’ end by SARS-CoV-2 non-structural proteins, allowing the virus to efficiently escape recognition of the viral dsRNA by the host cell sensor [77]. Viral proteins inactivate key intermediaries in the IFN signaling cascade. A recent study showed that the SARS-CoV-2 ORF9b interacts with MAVS in mitochondria, resulting in a decrease in TRAF3 and TRAF6 [78]. The nsp13 and nsp15 proteins of SARS-CoV-2 interfere with TBK-1 signaling and activate IRF3 [78]. Another key virulence factor for SARS-CoV-2 is Nsp1, which inhibits the expression of the host gene; thus, it can effectively block the innate immune responses which could assist in the eradication of infection [71]. The right panel shows that the viral protein Nsp3 blocks IFN signaling by reversible post-translational modification of ISG 15 [79]. In patients with severe COVID-19, a significant mitigation of IFN I response is associated with a clinically persistent viral load and increased oxidative stress and an inflammatory response [70]. IFN: interferon; ISGs: interferon-stimulated genes; MAVS: mitochondrial antiviral signaling protein; nsp: nonstructural protein 13; TRAF: tumor necrosis factor receptor associated factor. Figure generated with Biorender (https://biorender.com/accessed on 6 September 2021).

An earlier report revealed elevated inflammatory cytokines and IFNs in bronchial alveolar lavage (BAL) in patients with severe COVID-19 [66]. Although robust ISG induction by IFNs was noted, no obvious modulation of IFN-I was observed in BAL cells [67]. Replication of SARS-CoV-2 also resulted in decreased levels of IFN-I and IFN-II in a cellular model and in postmortem lung tissue of patients with COVID-19 [68]. It has also been reported that in ex vivo human lung tissue explants, SARS-CoV-2 did not significantly induce IFN-I [69]. In patients severely affected by COVID-19, an elevated viral load has been reported to be associated with a decreased IFN-I response [70] (Figure 2).

SARS-CoV-2 Nsp 1 (non-structural protein 1) can interfere with innate immune responses through RIG-I-dependent IFN-I/III production and virus removal. [71]. Because replication of the coronavirus RNA occurs in a particular double-membrane vesicle (DMV) that separates the virus-related PAMP from the cytoplasmic RNA sensor, this compartmentalization also reduces the ability of the cytoplasmic sensor to detect virus replication [72]. Finally, activating NF-κB significantly weakens IFN-I signaling, thus promoting viral replication [73], indicates that a cross-regulatory function exists between the IFN-I/III and the NF-κB signaling cascades [74]. NK cells are lymphocytes that play a significant role in the innate antiviral immune response. A recent in vivo study demonstrated that RSV activated NK cells and improved IFN-γ production and the cytotoxicity of NK cells, particularly in the presence of interleukin-2 (IL-2) [75].

### 4.3. Neutrophils and NETs

**Figure 3:**
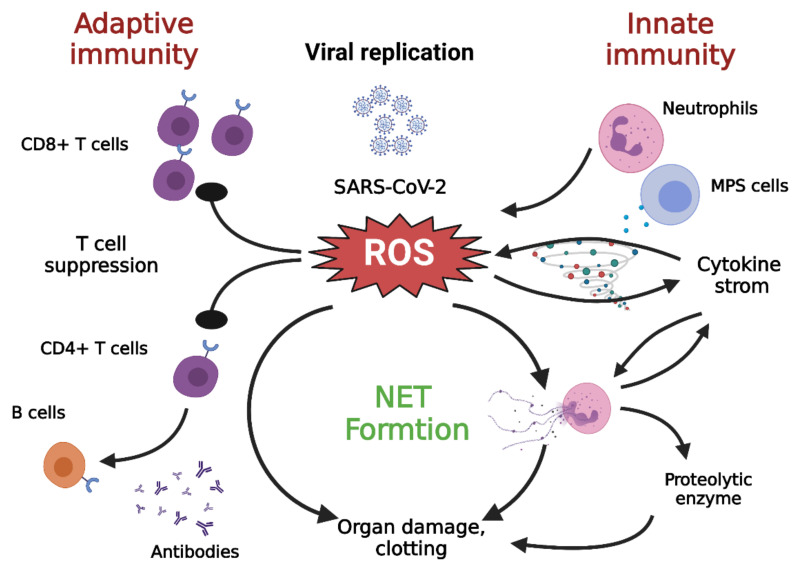
Cross talk among NETs, oxidative stress, and T cell deficiency [52]. The immune pathogenesis of COVID-19 includes both innate and adaptive immune systems. As the virus escapes IFN-I/III surveillance, the long-term, large-scale replication of the virus is initiated in host pulmonary epithelial cells, monocyte/macrophages, and vascular endothelial cells. As a result, neutrophils and MPS cells are recruited in large numbers into inflamed tissues. T cells can kill infected cells and eventually eradicate the virus. In addition, CD4+ T cells are less effective in promoting B cells to generate neutralizing antibodies and develop a long-lasting immune response. DAMP: damage associated molecular pattern; IFN-I/III: interferon I/III; MPS, mononuclear phagocytic system; NETs, neutrophil extracellular traps; ROS: reactive oxygen species; TLR: toll-like receptor. Figure generated with Biorender (https://biorender.com/accessed on 6 September 2021).

Neutrophil extracellular traps (NETs) contributes to inflammation. When pathogens are present, circulating neutrophils release granular proteins and chromatin to form extracellular fibrous matrices called NETs. NETs bind to pathogens and neutralize them, undergoing a process of cell death called NETosis [80]. Several enzymes participate in the formation of NETs, such as neutrophil elastase (NE) [81,82], peptidyl arginine deiminase type 4 (PAD4) [83], and gasdermin D [84]; these enzymes facilitate leakage of the cell membrane and the expulsion of its DNA and related molecules (Figure 3). 

While NETs promote host immunity against pathogens, the collateral damage caused by persistent NET formation is exacerbated in many disease processes [85]. NETs can facilitate macrophages to secrete IL1β, which further enhances the formation of NETs [86,87]. Furthermore, excessive NET formation can trigger inflammatory reactions that promote the destruction of surrounding tissue and cause permanent damage to the lungs and vital organs [88]. Furthermore, oxidative stress not only increases the formation of NETs but also contributes to the suppression of T lymphocytes [52]. In addition, IL-1β can promote IL-6 expression, which in turn induces fibrin release and fibrinogen expression, while the virus-induced endothelial damage can expose tissue factors, all of which accentuate the coagulation pathway, which further aggravates fibrin deposition and clotting [16].

### 4.4. Contribution of Monocytes/Macrophages to Oxidative Stress

Zhou et al. found significantly increased circulating CD14^+^CD16^+^ monocytes in hospitalized patients with COVID-19, a characteristic that was substantially prominent in patients with acute respiratory distress syndrome (ARDS). This finding suggests a certain degree of differentiation of monocytes to macrophages during SARS-CoV-2 infection. Circulating monocytes/macrophages express high levels of cytokines, such as IL-6, TNFα, and IL-10. This explains the relationship of these cells with oxidative stress, cytokine storm, and increased disease severity [20].

Class II histocompatibility complexes, such as HLA-DR, are responsible for presenting antigens to CD4^+^ T helper cells. The aberrant regulation of HLA-DR on monocytes and antigen-presenting cells, as is seen in cases of COVID-19, can result in systemic inflammatory conditions [89].

## 5. Adaptive Immunity in COVID-19

An effective long-term antigen-specific immune response is key to controlling long-term viral infections and preventing viral persistence. Indeed, all individuals who have recovered from COVID-19 carry virus-specific CD4^+^ T cells [90], which is strongly indicative of a crucial role for the immune response in the successful elimination of SARS-CoV-2. This adaptive immunity is suppressed in elderly patients [91] which could partially be because of defective monocyte-T cell crosstalk. Severe COVID-19 has also been associated with a higher frequency of depleted T cells [92]. T lymphocyte depletion has been widely described as senescence [93], further supporting the monocyte dysregulation, damage to adaptive immunity, and the severe COVID-19 seen in elderly patients (Figure 3).

### 5.1. Antigen-Specific Immunity Provided by the Adaptive Immune System

During viral infections, CD8^+^ cytotoxic T cells play a vital role in eliminating infected cells, while CD4^+^ T helper cells assist CD8^+^ T and B lymphocytes in generating neutralizing antibodies that are critical for viral eradication and long-lasting immunity. IFNs produced by CD4^+^ and CD8^+^ T cells exert antiviral effects by inducing the activation of ISGs, thereby promoting antiviral immunity [94]. The adaptive immune system appears to be a key factor in tissue healing processes. For example, regulatory T cells (Treg) were demonstrated to promote the repair and regeneration of different organ systems [95]. The presence of interleukin-10 (IL-10) improves the activity of Treg cells, which can lead to immune tolerance and suppression. Tregs also combat innate immune-induced inflammation. They prevent the production of IL-6 by neutrophils and stimulate them to produce anti-inflammatory mediators [96]. By releasing TGF-β, Tregs can induce neutrophilic apoptosis, which could help solve the pathology characteristics of acute lung lesion [97]. Tregs may also inhibit the infiltration of neutrophils into damaged tissues [98]. Therefore, it is possible to assume that Tregs are important for tissue regeneration in COVID-19.

### 5.2. Failure of Antiviral T Cell Responses

Viral infections are generally associated with lymphocytosis caused by the expansion of antigen-specific CD8^+^ T cells. However, in COVID-19, there is a decrease in CD4^+^ and CD8^+^ T cell numbers and a delayed and limited production of γ-IFN by CD4^+^ T cells [99,100]. Severe COVID-19 often presents with lymphopenia with increased neutrophil counts [99,101]. The neutrophil-lymphocyte ratio was found to be a critical risk factor associated with severe COVID-19 progression [102,103,104].

Persistent exposure to viral antigens causes exhaustion of T cells [105]. Exhausted T cells (Tex) express programmed cell death protein 1 (PD-1) or T-cell immunoglobulin and mucin domain 3 (TIM-3), which triggers T-cell apoptosis. Indeed, the subacute progression of COVID-19 is due to the depletion and exhaustion of T cells, representing a failure of adaptive immunity [106]. Clinically, T-lymphocytes from COVID-19 patients express high levels of PD-1 and TIM-3 [92]. The resulting low adaptive immunity environment allows for the continuous replication of the virus, which stimulates immunopathogenesis through the excessive inflammation of innate immune cells [107]. The precise mechanism that contributes to antiviral T cell deficiency in response to COVID-19 is unknown.

### 5.3. Oxidative Stress Suppression of T Cells

Apoptosis is one of the crucial mechanisms by which the reduced T-cell count associated with COVID-19. Chu et al. demonstrated that MERS-CoV can infect human T cells and trigger T cell apoptosis [108] by initiating Fas signaling [109]. A reduction in the number of T lymphocytes may also be caused by oxidative stress [110], which is generally present in patients with COVID-19 [111]. Pro-oxidative setting results were reported in the oxidation of T cell regulatory proteins, which results in hyporeactivity or even the death of T cells [111,112].

Furthermore, the SARS-CoV papain-like protein substantially activates the MAPK/STAT3 and ROS/p38 signaling pathways, which leads to an upregulation of TGF-β1 promoter expression in pulmonary epithelial cells. This results in the augmentation of pro-fibrotic responses [113]. Because TGF-β is a potent immunosuppressant affecting T lymphocytes, the release of ROS-mediated TGF-β can also contribute to the depletion of lymphocytes in COVID-19. Finally, maintaining T cells in a pro-oxidative state was shown to promote the development of Treg lymphocytes [114]. This relative increase in Tregs can also affect the ability of T cells to defend against a SARS-CoV-2 infection [102].

## 6. Aging in COVID-19

The number of deaths caused by the current COVID-19 pandemic is highly skewed toward older adults. Regardless of the country and stage of the outbreak, the death rate for COVID-19 increases exponentially with age [115]. Immune senescence is thought to be a determining factor in SARS CoV-2 infections [115,116]. 

Aging is associated with high levels of basal proinflammatory cytokines and acute phase proteins. The increased production of pro-inflammatory molecules and “inflamed aging” play a critical role in the development of the cytokine storm in patients with severe COVID-19 [117,118]. SARS-CoV-2 triggers a more potent NF-kB innate immune response in older animals than in younger animals, which results in an increased risk of ARDS [74]. This effect is associated with age-related increase in ROS levels, which reflects a compensatory homeostasis reaction to support physiological cellular signaling in older people [119]. The increase in ROS generation in older people further increases and reaches a threshold, causing NF-κB hyperactivity and inflammatory tissue injury [120]. The delayed and inadequate IFN-1 response to SARS-CoV-2 allows sustained virus replication and heightened oxidative stress, which triggers an NF-κB-induced cytokine storm and excessive inflammation [121,122]. Patients with COVID-19 have been shown to have upregulated NKG2A exhaustion markers in NK cells and CD8^+^ cytotoxic T cells [121]. In addition to the cytokine storm, SARS-CoV-2 can also directly damage multiple organs [123] (Table 1). 

### 6.1. Impaired IFN I Induction during Aging

**Figure 4:**
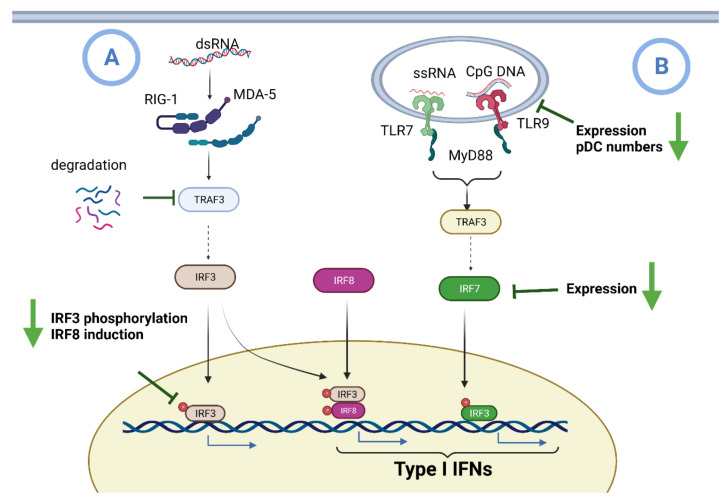
Impaired induction of IFN in aging. Respiratory epithelial cells, pDCs, cDCs, and macrophages/monocytes all have the ability to generate IFNs. (**A**) Recognition of viral dsRNA within infected cell cytoplasm by RIG-1/MDA-5 facilitates TRAF-3 to activate IRF3. Aging is associated with the degradation of TRAF-3 and decreased phosphorylation of IRF3. IRF3 acts as an intermediary for the transcription of IFN I and IRF8. IRF8 assists in amplifying the expression of IFN I. (**B**) The identification of viral rRNA and CpG DNA in pDCs by the intracellular double membrane vesicle containing TLR7 and 9 promotes the activation of MyD88 and TRAF6. In turn, this leads to the activation of IRF5 and IRF7, which translocate to the nucleus to promote the transcription of IFN I. Senescence reduces the number of circulating pDCs, the expression of TLR7/9, and the IRF7 adaptor expression [2]. IFN: interferon; IRF: interferon regulatory factor; cDCs: classic dendritic cells; pDCs: plasmacytoid dendritic cells; dsRNA: double-stranded RNA, MDA-5: melanoma differentiation-associated protein 5; RIG-1: retinoic acid-inducible gene 1; TLR: toll-like receptor; TRAF: tumor necrosis factor receptor associated factor. Figure generated with Biorender (https://biorender.com/accessed on 6 September 2021).

IFN I is largely induced by the recognition of viral components by RIG1/MDA-5 intracellular receptors and toll-type receptors (TLRs TLR3, 7, 8, and 9) [124]. TLR7 and 9 signaling induces IFN I expression through MyD88 and TRAF6 adaptors, resulting in the activation of the interferon regulatory factor 7 (IRF7) transcription factor [125]. In addition, the LRT cell membrane proteins, which are generally associated with bacterial pathogen recognition, may also induce IFN I generation in response to various viral infections [126,127]. Plasmacytoid dendritic cells (pDCs) are known for their robust ability to produce IFN I upon stimulation through TLR7 and TLR9 [128]. These cells are distinguished from classical DCs (cDCs) through their high expression of TLR9 and other markers such as B220 (CD45R), PDCA-1 (BST-2), BDCA-2, and BDCA-2 [129]. Aging is associated with the inability of monocytes and dendritic cells (DCs) to generate IFN in the presence of a viral infection, causing a dysregulation of the inflammatory immune response [2,130] (Figure 4).

### 6.2. Aging and ACE2 Receptor

The renin-angiotensin system consists of two opposite physiological homeostasis pathways including the ACE/Ang II/AT1 pathway, which is involved in tissue damage, inflammatory changes, and fibrosis [131,132], and the ACE2/Ang 1–7/Mas pathway, which exerts anti-inflammatory and anti-fibrotic effects [133]. Human monocytic cells and macrophages can express ACE2 [134], which makes them susceptible to direct SARS-CoV-2 infection. In hACE2 transgenic mice inoculated with SARS-CoV-2, viral antigens could be detected in mouse alveolar macrophages. This provides evidence of the ability of the virus to infect these cells directly [135,136].

A study by Xudong et al. revealed a significant reduction in ACE2 expression with aging, which may cause the pathogenesis of SARS infections in older patients [137]. An analysis of GTEx gene expression data revealed remarkably high expression levels of ACE2 in Asian females, a prominent decrease in obesity-related diabetes, and an age-dependent decrease in ACE2 expression [138]. These results indicate that the elevated expression of ACE2 in lung epithelial cells in children and young adults may have a protective clinical effect. As a result, low ACE2 expression levels and an imbalance of Ang II/Ang1–7 during aging are strongly associated with the cytokine storm, oxidative stress, and pulmonary lesions [139].

### 6.3. Aging and Excess Production of ROS

The mitochondrial respiratory chain and NADPH oxidase are the two major sources of ROS [140]. Pre-maturation aging mice have low antioxidant levels, but high levels of ROS and pro-inflammatory cytokines in immune cells [141]. The generation of surplus ROS seen with aging can initiate an inflammatory process through transcription factors linked to the immune system [142] was reported, and subsequently, can increase the synthesis and release of pro-inflammatory cytokines. Moreover, the additional production of ROS during aging upregulates the expression of transcription factor NF-κB. The generation of ROS leads to the inhibitory phosphorylation of IkB proteins, which promotes the nuclear translocation of NF-κB and an increase in the transcription of NF-κB responsive genes. Both activated NF-κB and excessive ROS production lead to an increase in the release of pro-inflammatory cytokines [6,142]. Hence, the additional production of ROS plays a role in the pathogenesis of chronic inflammation and “inflammatory aging” in older adults.

### 6.4. Aging and Immune Senescence

Immunosenescence is defined as the continuous weakening of immune effectors during aging [143,144,145]. Macrophages play an important role in the innate immune system [146,147]. The levels of several macrophage-induced factors and the expression levels of nitric oxide synthase in macrophages are clearly reduced during aging. Monocyte/macrophage phagocytic and chemotactic activity and the NK cell production of IFNs are both decreased with advancing age [148,149] (Table 2). In addition, NK cell phenotypes are altered and the expression of the CD57^+^ immunity-senescence marker is increased during aging [150,151,152]. High levels of age-related pro-inflammatory cytokines can induce the maturation and activity of DCs. An earlier study showed that there is an increase in the levels of pro-inflammatory cytokines released by DCs in older adults [153], with higher DC activity compared to young individuals [154]. Alterations in T-cell immunity have also been observed during aging [155]. DCs undergo continuous changes throughout their lives, and older DCs have been shown to have a reduced capacity to stimulate T cells. [156]. In humans, at birth, nearly all T lymphocytes express CD28^+^, but the proportion of CD28^+^ T cells decreases with age [157]. In physiology condition, CD28^+^ cells are linked to pro-inflammatory cytokine production and subsequently resist apoptosis [158,159,160].

Aging also alters the Th2 cytokine profile (IL-4 and IL-10) more than type Th1 (IL-2, IFN-γ). Pro-inflammatory Th17 cells are in balance with T_reg_ anti-inflammatory cells [161]. An increase in the Th17/Treg cell ratio leads to the development of a pro-inflammatory state in elderly patients [162,163]. Aging is also associated with a disruption in the regulation of lymphocytic telomerase [164], which causes shortened telomeres that are associated with age-related pathologies [165,166,167]. Aging is also accompanied by a significant reduction in the production of B cells in the bone marrow, but there is no decrease in the number of peripheral B cells. Owing to significantly reduced production with increasing age, B cells are selected based on their reactivity to environmental antigens and accumulate and present activated phenotypes [168,169]. Decreased T-dependent B-cell function during aging has been attributed to a lack of adequate T-helper capabilities [162]. Thus, the immune system appears to remain in a mild basal inflammatory state in older adults. However, the slightly overactive immune system in older adults cannot stop the pro-inflammatory state seen in SARS-CoV-2 infections and can result in serious cases. [150].

### 6.5. Aging and Vitamin D

Vitamin D (vit D) is an essential hormone supplied either as result of exposure to sunlight or via the ingestion of food. Most nucleated cells, including kidney tubule cells, contain 1-α-hydroxylase, which catalyzes the conversion of 25(OH)D_3_ into the active form of vit D (1,25(OH)_2_D_3_) [170]. In most immune cells, circulating 1,25(OH)2D3 is transported to the nucleus, where it binds to the vitamin D receptor (VDR). As a result, vit D can affect the transcription of many genes [171]. Macrophages increase VDR and 1-α-hydroxylase expression during a pathogenic challenge [172]. Vit D may suppress ROS production, expand intracellular glutathione pools, and suppress the expression of NF-κB and p38 MAP kinase to limit the expression of pro-inflammatory cytokines. Activation of TLRs or NLRs can increase intracellular VDR levels and 1-α-hydroxylase activity. Consequently, the complex of vit D and VDR can produce antimicrobial products such as cathelicidin and β-defensin in macrophages [173]. In addition, vit D can inhibit differentiation/migration and suppress the expression of MHCII in DCs [174]. However, elderly people are usually prone to have a vit D deficiency because of impaired pre-vit D production, inadequate skin integrity, low dietary intake, a high prevalence of increased adiposity, diminished renal function, and fewer outdoor activities [175,176]. Ilie et al. identified a negative relationship between average vit D levels and COVID-19 incidence and mortality in many countries. They found that vit-D levels are extremely low in the aging population, which is the most vulnerable population associated with SARS-CoV-2 infection [177]. Vit-D deficiency during aging also contributes to the upregulation of Th1 cytokine genes. Polymorphisms in the VDR gene, such as FokI, ApaI, and TaqI, are associated with vit-D deficiency, which can lead to a higher risk of inflammatory diseases [178]. Since the vit D and VDR pathways together perform a crucial anti-inflammatory role, vit-D insufficiency in older individuals likely increases the risk of chronic mild inflammatory conditions [176]. 

### 6.6. Phenotypes of Monocytes in Aging

The cellular functions of monocytes and macrophages are dysregulated during aging. A main feature seen in monocytes in elderly individuals is a higher proportion of unconventional monocytes, as well as a lower number of circulating conventional monocytes [179,180,181,182,183,184,185]. Compared with younger adults, monocytes derived from older adults show increased basal levels of cytokine production [182]. This can contribute to age-related chronic low-grade inflammation [186,187,188,189]. Aged monocytes exhibit reduced mitochondrial function [184], impaired phagocytosis [182], and reduced HLA-DR expression [181]. The effect of aging on monocytes includes an increase in circulating CD16^+^ monocytes, a decrease in IFN I production, and a decrease in efferocytosis (i.e., the process by which apoptotic cells are removed by phagocytic cells) [190]. Aging induces a change in the proportion of CD16^+^ subpopulations, in line with the monocyte phenotypes observed in severe COVID-19 cases. Therefore, monocyte dysfunction could play a crucial role in increasing the severity of COVID-19 pathogenesis in older people [191,192,193,194].

### 6.7. RBCs and Oxidative Stress in Aging

Because of the discrepancy between antioxidants and pro-oxidants, RBCs could be an important source of the oxidative stress seen with aging. Oxygen radicles from RBCs are generated by the decay of heme. The non-enzymatic degradation process is initiated by hydrogen peroxide, whereas the enzymatic route is mediated by heme oxygenase-1 (HO-1). Consequently, biliverdin, carbon monoxide (CO), and iron ions (Fe^2+^) are generated, contributing to the oxidative processes in erythrocytes [195]. The non-enzymatic degradation of heme causes the accumulation of ROS in RBCs resulting in changes in membrane structure and function. This causes a loss of membrane integrity and reduced RBC deformability. All these changes affect the function of RBCs in hemostasis, thrombosis, and accelerated aging [196,197,198].

#### COVID-19 and Heme Oxygenase

**Figure 5:**
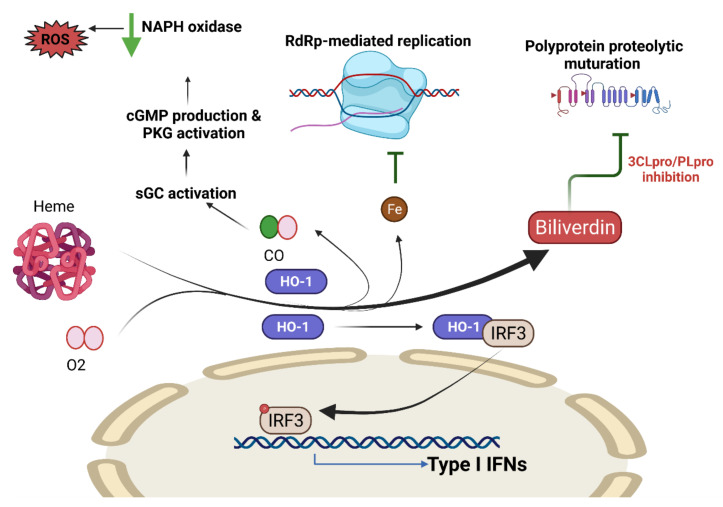
Nrf2-targeted HO-1 antiviral activity. Nrf2-targeted HO-1 catalyzes the enzymatic degradation of heme into CO, Fe^2+^, and biliverdin. CO activates sGC to generate cGMP, which allows PKG to inhibit ROS production via NADPH oxidase (NOX), which can prevent the exacerbation of oxidative stress. Free Fe^2+^ released from heme binds to the viral RdRp divalent metallic binding site to inhibit viral replication. By reducing the activity of the 3CLpro and PLpro proteases encoded by SARS-CoV-2, biliverdin prevents viral peptides from maturing. The heterodimerization of HO-1 and IRF3 promotes the phosphorylation and nuclear translocation of IRF3, which drives IFN I gene expression. CO: carbon monoxide; HO-1, heme oxygenase 1; IFN, interferon; ISRE, interferon-sensitive response element; IRF3, interferon regulatory factor 3; NRF2, nuclear factor erythroid 2 p45-related factor 2; P, phosphorylation; PKG, protein kinase G; sGC, soluble guanylate cyclase; RdRp, RNA-dependent RNA polymerase. Figure generated with Biorender (https://biorender.com/accessed on 6 September 2021).

The cytoprotective properties of HO-1 are due to its ability to cleave heme into biliverdin, Fe^2+^, and CO, thereby providing a tissue protective effect as a result of the attenuation of inflammation and oxidative stress [199,200,201]. COVID-19 prevents the synthesis of heme, which limits the stress reducing effects of HO-1, thus contributing to host illness. A previous report in Taiwan found high levels of HO-1 expression in subjects with the HO-1(497A/*) genotype, which protects against SARS-CoV-2 infection [202] (Figure 5).

Abouhashem et al. showed that there was a significant reduction in HO-1-induced gene expression in the tissues of older individuals with low levels of HO-1, which resulted in a high inflammatory status [203]. It has been reported that HO-1 has antivirus activity [204]. As a result, COVID-19 could end the production of HO-1, which is consistent with heat shock proteins (Hsps) being a threat to the virus [205]. Viral infections may induce an inadequate response to cell stress, thereby reducing stress tolerance and making tissues vulnerable to damage.

Some studies used hyperbaric oxygen therapy (HBOT) to increase oxygenation for COVID-19 patients [206]. HBOT enhances tissue HO-1 levels and allows it to exert its cytoprotective effects [207]. Our previous study showed that RSV increases the expression of HO-1 and improves membrane kidney function in a mouse model of kidney disease [201]. 

## 7. Resveratrol and COVID-19

### 7.1. Anti-Aging Interventions

While COVID-19 is frequently not lethal among the young, the death rate increases exponentially with age, especially in those with age-related illnesses. This suggests that age is probably a good predictor of mortality. At its deepest point, aging results from excessive abnormal cellular functioning, which explains why COVID-19 susceptibility is age-dependent and related to other age-related comorbidities. Anti-aging interventions, such as rapamycin, may slow the aging process and potentially reduce COVID-19 vulnerability [208]. The natural anti-aging compound RSV has positive effects on lifetime, age-related illness, and maintenance of health. The different major mechanisms modulated by RSV can prevent or treat several age-related diseases. Thus, RSV appears to have a similar effect to rapamycin, even though it acts through a different mechanism [209].

Aging physiology aims to understand how aging affects the body’s ability to withstand adverse events and how age-dependent molecular and cellular changes are involved in this process [210]. The age-related exaggerated immune responses to COVID-19 that are characterized by a “cytokine storm” [211] and intravascular coagulation [212] may be attributed to the fact that the capacity of the human body to regulate its responses to threats is increasingly compromised during aging.

As many as 95% of COVID-19 victims have certain aging-related conditions, the prevalence of which increases exponentially with age [196]. Thus, a potential measure to preventing or treating COVID-19 could be to utilize the methods that are generally suggested to alleviate the decline in age-dependent fitness and the associated development of age-related pathological processes.

### 7.2. Resveratrol as a Modulator of Antiviral Agent

Duck enteritis virus (DEV) is a double-stranded DNA virus belonging to the Alphaherpesvirinae sub-family of Herpesviridae [213]. A previous study confirmed that RSV significantly reduces the mortality of ducklings infected with a virulent strain of DEV. Pathological symptoms and viral loads in blood and tissue were decreased effectively compared to the untreated group. In this study, a low concentration of RSV increased the production of IFN-α, IL-2, and IL-12, while these cytokine levels decreased by a high concentration of RSV [214]. Rotavirus (RV) is a double-stranded RNA virus within the family Reoviridae. The gastroenteritis induced by RV, which is the foremost etiological agent for acute pediatric viral diarrhea worldwide, is a leading cause of death among children less than 5 years of age [215]. RSV could alleviate diarrhea, inhibit the production of TNF-α, and increase IFN-γ levels after RV infection [216]. Another study evaluating the efficacy of a nasal resveratrol/carboxymethyl-β-glucan (RSV/CMglucan) solution in infants with a common cold showed that it might have a positive impact on clinical outcomes [217]. Middle Eastern respiratory syndrome (MERS) is a viral respiratory disease caused by the MERS coronavirus (MERS-CoV) [218]. A study of MERS-infected Vero E6 cells showed that RSV significantly inhibited MERS-CoV infection and prolonged cellular survival after viral infection. Expression of the nucleocapsid (N) protein, which is essential for MERS-CoV replication, also decreased after RSV treatment. RSV also reduced apoptosis in the infected cells. Therefore, RSV may be useful as a treatment for MERS-CoV infections [219]. 

ACE2 and TMPRSS2 both expressed in human cornea epithelial cells (HCEC). ACE2 expression is upregulated in HCECs, following stimulation with TNFα and IL-1β, and in inflamed corneas. RSV could attenuate the increased expression of ACE2 induced by TNFα in HCECs [220]. With regard to the ACE2–spike interaction that occurs in COVID-19, molecular docking simulations provided evidence that RSV can bind to spike, ACE2, and the ACE2–spike complex with good affinity. Preliminary biochemical assays revealed a significant inhibitory effect of RSV on ACE2-spike binding [221]. A recent mini-review found that the antiviral efficacy of RSV for a number of viruses, including the coronavirus. RSV can modulate the major pathways involved in the pathogenesis of SARS-CoV-2, including regulation of the renin-angiotensin system (RAS) and the expression of angiotensin-converting enzyme 2 (ACE2), stimulation of the immune system, and downregulation of pro-inflammatory cytokines release. RSV can stimulate the SIRT1 and p53 signaling pathways and increase cytotoxic T lymphocytes and NK cells. In addition, RSV treatment has been shown to be a fetal hemoglobin stimulator and a potent antioxidant that traps ROS [222].

Various natural products have been found to be potential anti-COVID-19 agents. In this regard, it was suggested that RSV might be used alone or in combination with FDA-approved medications to treat COVID-19 [223]. The use of RSV in clinical practice is limited by low bioavailability following oral administration because of the pharmacokinetic and metabolic characteristics of the molecule. Therefore, topical administration through inhaled formulations, such as an aerosol, could allow the administration of sufficiently high concentrations of the compound through the airways, which are the entry route for SARS-CoV-2 [224,225]. RSV/CMglucan formulation may be appropriate for simultaneously volatilizing aerosols to treat patients with lower respiratory tract diseases [221]. This could potentially suppress viral replication in the early stages of infection, reducing viral spread in the lower respiratory tract, leading to a reduction in the risk of infection transmission. 

#### 7.2.1. NF-κB and Nrf2 in COVID-19

The NF-κB and Nrf2 signaling pathways play a significant role in oxidative stress and cytokine storms, which are the hallmarks of COVID-19 [226]. 

##### NF-κB and COVID-19

Many molecular mechanisms have been identified as the routes through which the antiviral effects of RSV are mediated, such as the regulation of critical cell pathways, including those involved in the cell cycle, apoptosis, and inflammatory responses [5]. It is known that RSV inhibits the NF-κB pathway, which is a major regulator of the inflammatory cellular response. It is also involved in cell proliferation and transformation [227,228,229,230]. 

The NF-κB transcription factor family is a pleiotropic master gene product of many cellular signaling pathways that stimulate inflammation. The hyperinflammation pathway could regulate NF-κB signaling and increase the levels of cytokines and other inflammatory mediators in patients with COVID-19 [231]. Phosphorylation of the inhibitory protein IκB during inflammation causes the release of NF-κB, allowing for its translocation to the nucleus. Therefore, NF-κB activation is a feature of inflammatory illness [232]. The cytokine storm is characterized by elevated levels of IL-6, IFN-γ, and TNF-α, as well as granulocyte colony-stimulating factor (G-CSF) [233]. While NF-κB activation is often protective, some viruses, such as the influenza A virus, rely on NF-κB activation to allow for their efficient replication [234,235]. Therefore, inhibition of the NF-κB pathway could be a mechanism through which RSV exerts its antiviral activity [236].

##### Nrf-2 and COVID-19

Nrf2 is a transcription factor that binds to its inhibitor Keap1 in the cytoplasm. When the cell is exposed to stress, electrophilic molecules or the production of ROS cause the dissociation of the Nrf2-Keap1 complex. Free Nrf2 is then translocated into the nucleus to stimulate the transcription of several genes involved in redox and antioxidant responses [237]. A study on the Nrf2 enhancer showed that it downregulated 36 genes that encode various cytokines, resulting in a reduction in the cytokine storm in COVID-19 [238].

Under conditions of oxidative stress, Nrf2 generates a variety of cytoprotective and antioxidant enzymes to counteract the oxidative stress [239]. The upregulated target genes include glutathione S-transferase, catalase, HO-1, and superoxide dismutase, all of which provide a protective function against oxidative stress. Nrf2 increases HO-1 expression and triggers heme catabolism via cleavage of the porphyrin ring to produce carbon monoxide, Fe^2+^, and biliverdin [240]. Nrf2 prevents NF-κB activation by increasing the expression of HO-1, thereby reducing cytokine release. Nrf2 also attenuates the degradation of IκB-α, thus inhibiting NF-κB-mediated transcription, further leading to the inhibition of the release of pro-inflammatory cytokines [239]. Therefore, the activation of Nrf2 may be advantageous in treating COVID-19 [241]. In this regard, RSV, an Nrf2 activator, may provide adequate protection in elderly patients with COVID-19.

##### Interplay between the Nrf-2 and NF-κB Pathways

**Figure 6:**
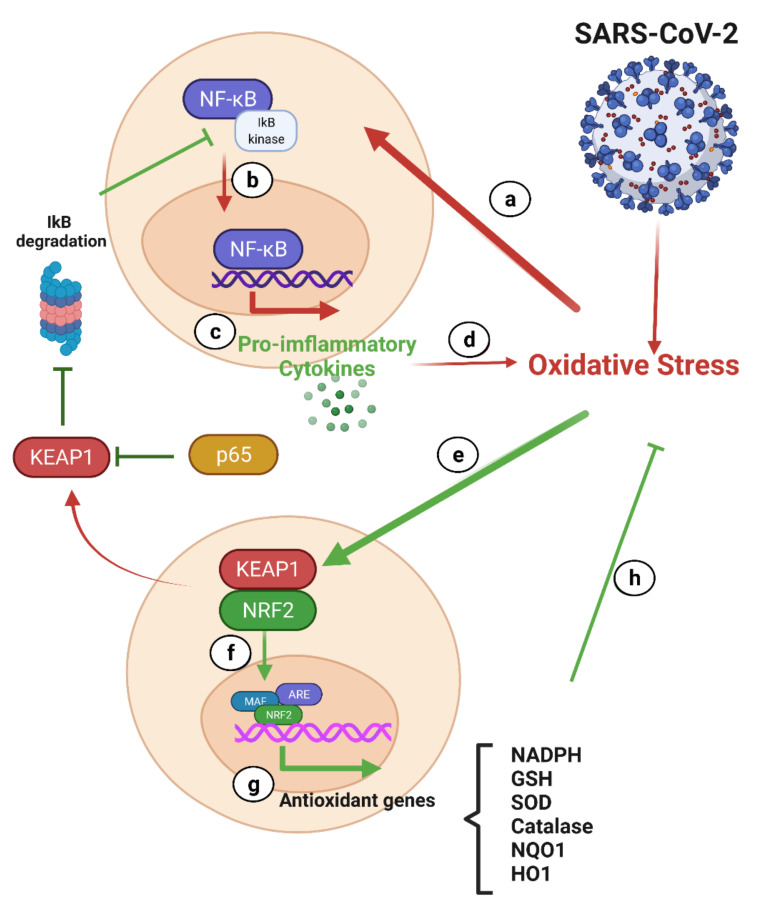
Crosstalk between NF-κB and Nrf2 signaling in COVID-19 [226]. In uninfected cells, the NF-κB subunit is limited to the cytoplasm because of the inhibitory effect of the κB inhibitor family (IκB). (**a**) SARS-CoV-2 infection can increase oxidative stress and cause IKβ kinase activation, which causes phosphorylation of IkB-α (an NF-κB inhibitor), leading to proteasomal degradation of IkB-α and subsequent release of NF-κB. (**b**) In infected cells, NF-κB containing p65 (a NF-kB subunit; KEAP1 inhibitor) is transferred to the nucleus and acts on the DNA response elements. (**c**) This leads to the transcription of numerous pro-inflammatory cytokines. (**d**) The activated NF-κB signaling cascade produces excessive pro-inflammatory cytokines and exacerbates the oxidative state. (**e**) In contrast, oxidative stress elicits Nrf2 signaling, leading to the separation of Nrf2 from its inhibitor Keap1. (f) Nrf2 then moves to the nucleus and binds to the Maf protein and the antioxidant response element (ARE). (**g**) Activated ARE transcripts include antioxidant genes and phase II enzymes such as NADPH, GSH, SOD, catalase, heme oxygenase-1, and NQO1. All these enzymes enhance the degradation of ROS. In addition, free Keap1 can prevent the degradation of IkB-α. (**h**) In general, genetic intervention and subsequent transcription have a positive effect of the Nrf2 pathway in the reduction of oxidative stress. Infection with COVID-19 causes oxidative stress, which also causes antioxidant benefits associated with Nrf2 [226]. Figure generated with Biorender (https://biorender.com/accessed on 6 September 2021).

In most tissues, cells have different levels of oxidative stress and inflammation. Nrf2 and NF-κB are the two main transcription factors that regulate cell responses to oxidative stress and inflammation, respectively. Nrf2 and NF-κB are regulated by redox sensitivity factors, and the absence of Nrf2 is associated with increased oxidative and nitrosative stress, leading to the amplification of NF-κB signaling [239] (Figure 6). There are several mechanisms by which p65 (the canonical subunit of NF-κB) can adversely affect gene expression associated with the antioxidant response element (ARE). Yu et al. demonstrated that p65 contributes to an increased abundance of nuclear Keap1 levels [242] and inhibits the Nrf2-ARE pathway, resulting in downregulation of expression of cytoprotective enzymes [243]. The well-established mechanism of p65 inhibition of Nrf2 activity leads to local acetylation of histones, loosening of the chromatin structure, and exposure of DNA, allowing for assembly of the transcriptional apparatus [240].

##### Effects of Resveratrol on the Nrf2 Signaling Pathway

**Figure 7:**
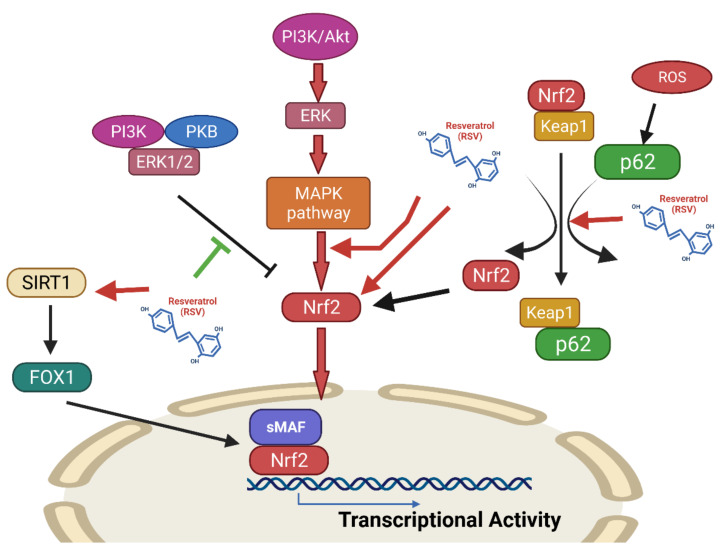
RSV increases the transcriptional activity of Nrf2. Regulation of the expression of antioxidant genes is critical for controlling oxidative stress and maintaining physiological homeostasis. Of the various regulatory pathways, the Keap1-Cul3-Rbx1 axis is the most important regulator of Nrf2 activity [252]. The Nrf2 pathway is essential for modulating the inflammatory responses and oxidative stress [253]. Mechanistically, RSV decouples the connection between Nrf2 and its inhibitor Keap1 by increasing the interaction between p62 and Nrf2 [254]. This leads to increased Nrf2 translocation into the nucleus, resulting in increased transcriptional activity [255]. Under normal physiological conditions, Keap1 blocks Nrf2 by retaining Nrf2 in the cytoplasm, thereby targeting it for proteasomal degradation. However, oxidative stress affects the structure of Keap1, making it incapable of inhibiting Nrf2 and retaining it in the cytoplasm [256]. Nrf2 may also be activated by the phosphatidylinositol 3-kinase (PI3K)/protein kinase B (Akt) signaling pathway, particularly in response to RSV [257]. RSV can also activate the Nrf2/ARE pathway by boosting p38 MAPK and SIRT1/FOXO1 signaling. Therefore, RSV enhances Nrf2 expression and potentiates Nrf2 signaling by changing Nrf2 mediators and its nuclear translocation through blockage of Keap1 [244]. Figure generated with Biorender (https://biorender.com/accessed on 6 September 2021).

The effects of RSV in reducing oxidative stress which are mediated by blocking Keap1 in order to enhance Nrf2 signaling, by increasing the activity of Nrf2 activators, enhancing its expression, and increasing its nuclear translocation [244]. RSV can potentiate Nrf2/HO-1 activity, activate SIRT1/AMPK signaling, enhance antioxidant defenses, and attenuate oxidative damage, in a periodontitis animal model [245,246]. RSV has also been shown to ameliorate oxidative damage in acute septic lung injury by modulating PI3K/Nrf2/HO-1 signaling [247]. In addition, RSV can suppress IL-18, IL-1β, IL-6, and TNF-α expression and attenuate the activity of caspase-3/9 in an osteoarthritis model [248]. In addition, RSV suppresses osteoarthritis by suppressing NF-κB and increasing Nrf-2/HO-1 activation [249] (Figure 7). 

**Figure 8:**
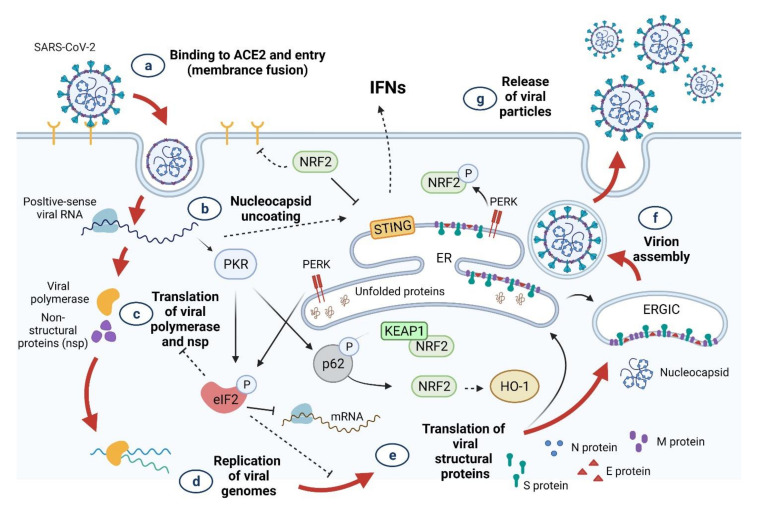
Potential crosstalk between the SARS-CoV-2 viral cycle and the RSV-mediated induction of Nrf2 activity. RSV supplementation activates the transcriptional activity of Nrf2, which can interface with the SARS-CoV-2 viral cycle. This figure shows the various stages of the viral cycle that could be influenced by Nrf2 signaling. (**a**) Binding of the viral spike protein (S) to ACE2 results in virion entry. Nrf2 suppresses the expression of the ACE2 gene [262]. (**b**) The viral nucleocapsid is uncoated in the cytoplasm of infected cells. (**c**) Next, the translation of viral +ssRNA and division of the products into different viral proteins occurs. Moreover, viral RNA within the host cell activates the cGAS DNA/RNA sensor, which transmits signals through the STING adapter [263], and facilitates the expression of type I and III IFNs [264]. Nrf2 suppresses IFN production by reducing STING expression [265]. (**d**) With respect to the replication of the SARS-CoV-2 genome, Nrf2 promotes HO-1 expression, generating Fe^2+^ that can bind to the divalent metal-binding pocket of the RdRp of SARS-CoV-2 and inhibits its catalytic activity [266]. (**e**) To allow for the translation of viral structural proteins, double-stranded RNA-activated PKR phosphorylates eIF2 and inhibits the translation of viral proteins. PKR also phosphorylates p62 and activates Nrf2 when its suppressor, KEAP1, is removed by autophagy [267]. Nrf2 is a PERK substrate that acts as a PERK-dependent cell-survival effector. Inhibition of viral proteins also activates the UPR. PERK is a vital Ser/Thr kinase protein that also plays a role in the UPR pathway. Nrf2 phosphorylation leads to the stabilization and improvement of its transcriptional activity [268]. (f) Virion assembly. Autophagy acts as a cell-monitoring mechanism for the control of invasive pathogens. SARS-CoV-2 ORF3a inhibits autophagy activity by blocking the formation of autolysosomes, which may destroy the newly synthesized virion [269]. The newly formed viral particles are assembled across the endoplasmic reticulum and the Golgi complex. (**g**) Release of viral particles. Structural proteins play an important role in the budding of viral particles released by infected host cells [270]. Numerous studies have proposed that ACE2 receptors are key structural proteins for virus budding and entry into host cells [271]. ACE2: angiotensin-converting enzyme 2; eIF2: eukaryotic initiation factor 2; ER: endoplasmic reticulum; ER: Golgi intermediate compartment; HO-1: heme oxygenase 1; IFN: interferon; KEAP1: Kelch-like ECH-associated protein 1; NRF2: nuclear factor erythroid 2 p45-related factor 2; PERK: PKR-like endoplasmic reticulum kinase; P: phosphorylation; PKR: protein kinase R; STING: stimulator of interferon genes; RdRp, RNA-dependent RNA polymerase; UPR, unfolded protein response. Figure generated with Biorender (https://biorender.com/accessed on 6 September 2021).

Natural antioxidants may be useful for treating age-related diseases [250]. In an aging mouse model, RSV was shown to have a protective effect against renal damage through the activation of Nrf2/HO-1 signaling. RSV attenuated mitochondrial ROS generation by activating Nrf2 in aged VSMCs [251]. This explains why Nrf2 inductors may be considered to prevent excessive inflammatory and oxidative responses in older patients with COVID-19 (Figure 8).

Both in vitro and in vivo experiments revealed numerous beneficial effects related to RSV usage [258,259]. RSV was proposed for use in patients with COVID-19 due to its ability to reduce inflammatory cytokine levels [3,260]. Furthermore, higher levels of ACE2 have a protective effect against SARS-CoV disease, and it is known that a large dose of RSV upregulates ACE2 receptors, suggesting that RSV is potentially beneficial in the treatment of SARS-CoV-2, especially in elderly patients [3,261].

##### The Nrf2-ARE (Antioxidant Response Element) Pathway

**Figure 9:**
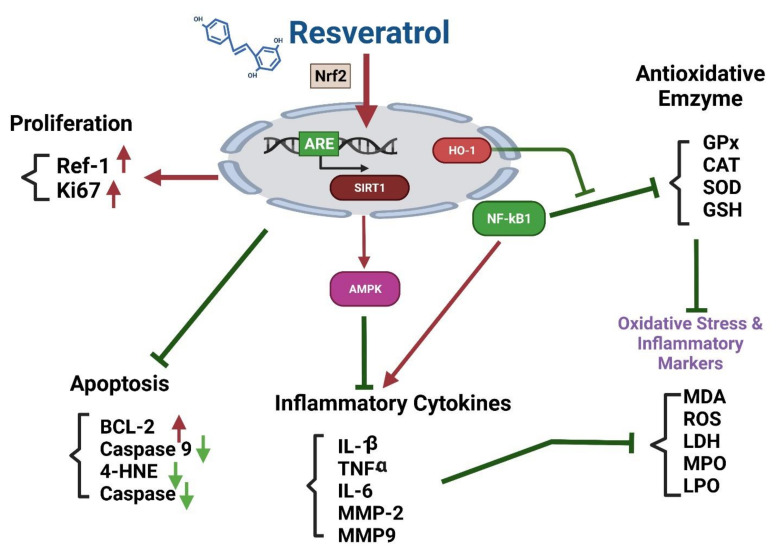
RSV activates the Nrf2/ARE pathway, which attenuates inflammation, oxidative stress, and apoptosis. Transcriptional activation of protective genes against SARS-CoV-2 infection is mediated by a cis-acting component called the antioxidant responsive element (ARE). From a molecular perspective, Nrf2 binds to ARE and subsequently activates this pathway to protect cells from oxidative stress [284]. RSV activates the Nrf2/ARE pathway and promotes the expression of HO-1, thus inhibiting the inhibitory effect of NF-Kβ on antioxidants and its stimulation of inflammatory flares. In addition, activating the Nrf2/ARE pathway increases the expression of SIRT1/AMPK, which leads to a decrease in inflammation. In short, activating the Nrf2/ARE pathway not only increases the resistance of cells to oxidative stress but also increases the expression of B-cell lymphoma 2 (Bcl-2; promotes cellular survival and inhibits the actions of pro-apoptotic proteins) and significantly inhibits Jun N-terminal kinase (JNK; activates apoptotic signaling by the upregulation of pro-apoptotic genes)-dependent caspase activity, thereby reducing host cell apoptosis. Figure generated with Biorender (https://biorender.com/accessed on 6 September 2021).

Nrf2 is a basic leucine zipper transcription factor that binds to the promoter sequence, referred to as ARE, leading to the increased expression of ARE-induced antioxidant genes, [272] such as those encoding phase II detoxification enzymes [273] and factors essential for the survival of several organs [274]. RSV has been shown to activate Sirt1 [275,276,277], and in turn, Sirt1 interacts with and deacetylates p53. This results in the suppression of p53-mediated functions [278,279] (Figure 9). Some studies demonstrated the inhibitory effects of RSV on cyclooxygenases 1 and 2 (COX 1 and 2), both pro-inflammatory enzymes [280]. Other studies reported its effects on apoptosis [281,282,283]. Whether virus infected cells undergo apoptosis, or whether apoptosis is suppressed, ultimately depends on the preferential activation of Sirt1 or p53 by RSV and the effects of RSV on the NF-κB pathway.

## 8. Conclusions

Elderly individuals are generally more likely to develop viral infections. COVID-19 is more severe among the elderly than youngsters, with a significantly increased mortality rate. Aged individuals often have levels of high basal inflammation and oxidative stress. COVID-19 leads to excessive activation of monocytes and macrophages. The delay and inadequacy of IFN production and signaling makes the detection and elimination of the virus difficult. This causes the virus to replicate in the body in large amounts, which further aggravates inflammation and oxidative stress. The viral proteins encoded by SARS-CoV-2 inhibit the production of IFNs and ISGs. Neutrophils may produce NETs and excess oxidative stress. In elderly patients, the response of T lymphocytes to the virus is noticeably inadequate, and the oxidative stress limits the normal differentiation of T lymphocytes. In addition, elderly patients often have many comorbidities, which further increase the degree of inflammation and oxidative stress, particularly upon SARS-CoV-2 infection. 

Elderly patients also face a significant decline in the ability to produce adequate IFN in the face of viral infections, as well as the performance of tissue/organ/cell ACE2. Older adults are also more likely to have vitamin D deficiency. COVID-19 may also result in changes in the morphology of monocytes, thereby affecting their function. Red blood cells contaminated with the virus also contribute to oxidative stress. For the holistic management of COVID-19, it is important to understand how to introduce anti-inflammatory therapies to complement antiviral therapy. Such therapies should treat innate inflammation without altering the host’s ability to elicit an aberrant adaptive immune response against the virus. Nrf2 activation, which promotes inflammation resolution, restores cell redox and protein homeostasis, and supports tissue repair, is a potential target for treating COVID-19. Furthermore, Nrf2 may be activated by pharmacological agents which target the KEAP1. Based on this information, RSV is likely a suitable choice, especially among older patients with COVID-19 [284].

What can we do to treat COVID-19 among elderly patients? It is particularly easy and practical to choose food supplements that are safe, inexpensive, and effective, which can reduce inflammation and oxidative stress. RSV has anti-aging, antiviral, anti-inflammatory, and antioxidant effects. Currently, the global prevalence of COVID-19 has not abated, and new virus variants are becoming more widespread. Considering the potential dangers for elderly patients, we believe that RSV offers suitable benefits. However, this requires further clinical investigation.

## Figures and Tables

**Table 1:** Comparison of alterations in innate immune responses during aging (inflammaging) and the absence/reduction in type I IFN [2,131].

Cell Type/Signaling	Aging	Absence/Reduction in Type I IFN
PRR activation & signaling	↑ High age-related basal PRR (TLR) activation leads to excessive pro-inflammatory cytokine production ↓ Post-PRR downstream signaling activation	↓ Recognition of intracellular pathogens ↑ Activity of Nlrp3 inflammasome ↓ ISGs expression signaling ↓Inducible nitric oxide synthase (iNOS)
Neutrophils	↑ Neutrophil influx through IL-17, CXCL1, CXCL2 ↓ Phagocytic function ↓ Signaling pathway	↑ Neutrophil recruitment by CXCL1 and CXCL2 production by monocytes
Monocyte/Macrophages	↑ IL-6 and TNF production ↓ Macrophage phagocytosis of apoptotic neutrophils ↓ Alveolar macrophage affects repair of lung damage	↓ Inflammatory monocyte-derived macrophages (IM) recruitment ↑ Resident IM proliferation ↓ IM iNOS, ↑ Ly6Clo monocyte iNOS production ↓ IM TRAIL expression ↓ Macrophage phagocytosis and efferocytosis of apoptotic neutrophils
NK cells, Type I IFN	↓ Delayed type I IFN activation and production ↓ Cytotoxic early viral clearance ↑ Cytokine production and consequent lung damage ↑ NK cell apoptosis	↓ NK cell activation and IFN-production ↓ NK cell survival
Dendritic cells	↓ Functional capability ↓ DC maturation and migration to lymphoid organs affect T cell activation	↑ cDC2 subtype development ↓ Antiviral responses by lowering ISGs ↓ Migration function

**↓**: decrease; **↑**: increase.

**Table 2:** Comparison of changes in adaptive immunity during aging (immunosenescence) and the absence/reduction of type I IFN [2,131].

Cell Type	Aging	Absence/Reduction in Type I IFN
**DCs**	↓ DC maturation and priming of T cells ↑ PD-L1/2 on cDCs	↓ moDC maturation and IL-12 production ↓ cDC maturation and priming of T cells
**T cells**	↓ CD4^+^ and CD8^+^ T cell expansion/survival ↑ Rapid activation of CD8^+^ T cell, high proliferation, and function resultant rapid fatigue ↓ Sensitivity to IFN-I signaling ↓ CD4^+^ memory T cells ↓ Memory T cell contraction ↑ PD-1 expression ↑ Pro-inflammatory Th17 cells ↓ Anti-inflammatory Treg suppression Initial low Th1/Th2 ratio leads to high viral titers and rapid switch to high ratio which leads to cytokine storm	↓ CD8^+^ and CD4^+^ T cell expansion/survival ↓ CD8^+^ T cell maturation and activation ↓ CD8^+^ memory T cell formation ↓ CD8^+^ memory T cell cytotoxic function ↓ Memory T cell contraction ↑ PD-1 expression and exhaustion of CD8^+^ T cells
**B cells**	↓ Vaccine seroconversion ↓ B cell proliferation ↓ T-bet expression ↓ Isotype switching ↓ Affinity maturation ↓ Antibody affinity	↓ B cell proliferation ↓ Plasma cell differentiation and antibody secretion ↓ T-bet expression ↓ Isotype switching ↓ Production of IgG2a, IgG1, IgG2b, and IgG3 antibodies
**T and B Memory cells**	↑ Tissue-specific-antibody experienced memory cells ↓ Naïve lymphocyte	↓ IgG1^+^ and CD86^+^ memory B cells ↓ Transcription factor Bcl-6 within Tfh cells

**↓**: decrease; **↑**: increase.

## Abbreviations

ACE2, angiotensin-converting enzyme 2; CAT, catalase; cDCs, classic dendritic cells; dsRNA, double strain RNA; eIF2, eukaryotic initiation factor 2; ER, endoplasmic reticulum; ERGIC, ER–Golgi intermediate compartment; GPx, glutathione peroxidase; GSH, Glutathione; HO-1, heme oxygenase 1; IFN, interferon; IRF3, interferon regulatory factor 3; ISG, interferon-stimulated gene; ISRE, interferon-sensitive response element; KEAP1, Kelch-like ECH-associated protein 1; LPO, lipid peroxidation; MDA, Malondiald; MMP, matrix metalloproteinase; MPO, Myeloperoxidase; MPS, mononuclear phagocytic system; NETs, Neutrophil extracellular traps; Nrf2, nuclear factor erythroid 2 p45-related factor 2; pDCs, plasmacytoid dendritic Cells; PERK, PKR-like endoplasmic reticulum kinase; PKG, protein kinase G; PKR, protein kinase R; ROS, Reactive Oxygen Species; sGC, soluble guanylate cyclase; SOD, Superoxide dismutase; ssRNA, single strain RNA; STING, stimulator of interferon genes; TRAM, TRIF-related adaptor molecule.
